# Necrosectomy performed by a single-use endoscope with a large operative working channel

**DOI:** 10.1055/a-2598-3566

**Published:** 2025-06-13

**Authors:** Alessandro Fugazza, Marco Spadaccini, Alessandro De Marco, Faisal Abubaker, Matteo Colombo, Cesare Hassan, Alessandro Repici

**Affiliations:** 1 Division of Gastroenterology and Digestive Endoscopy, Humanitas Research Hospital – IRCCS, Rozzano, Italy; 2 Department of Biomedical Sciences, Humanitas University, Pieve Emanuele, Italy; 3 King Hamad University Hospital, Al Sayh, Bahrain

A 61-year-old man with a large infected peripancreatic walled-off necrosis after acute pancreatitis 1 was managed with endoscopic ultrasound-guided placement of a lumen-apposing metal stent (20 × 10 mm Hot-Axios; Boston Scientific, Marlborough, Massachusetts, USA) for drainage 2 3 , followed by pneumatic dilation of the stent up to 15 mm and endoscopic necrosectomy ( Fig. 1 , Video 1 ). After necrosectomy, two double-pigtail plastic stents, 7 Fr × 7 cm and 7 Fr × 5 cm, were placed with a stent-in-stent technique ( Fig. 2 ). For necrosectomy we used a single-use endoscope with a large operative channel (4.2 mm) ( Fig. 3 ).

**Fig. 1:**
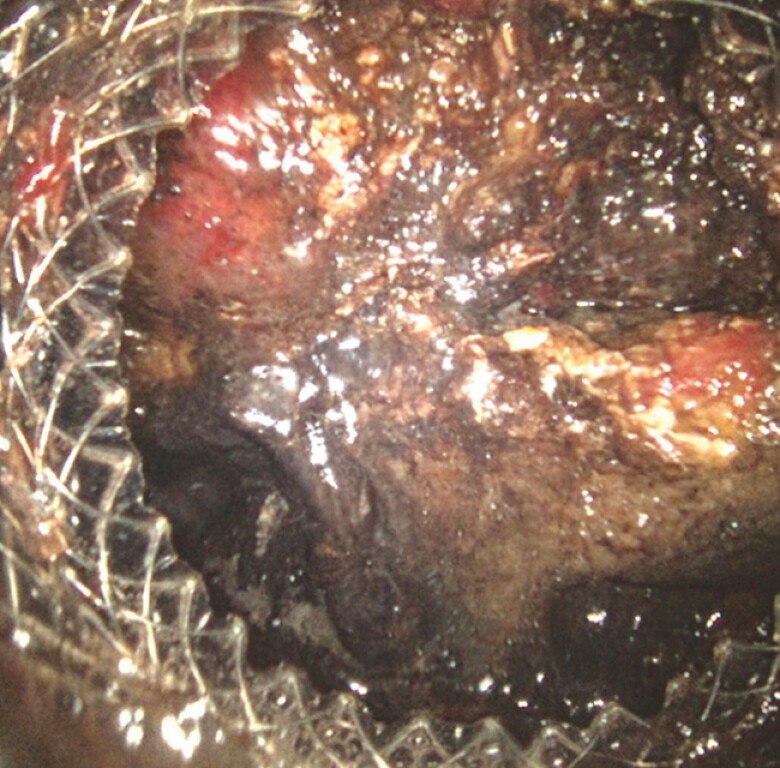
Direct endoscopic necrosectomy for management of walled-off necrosis.

**Fig. 2:**
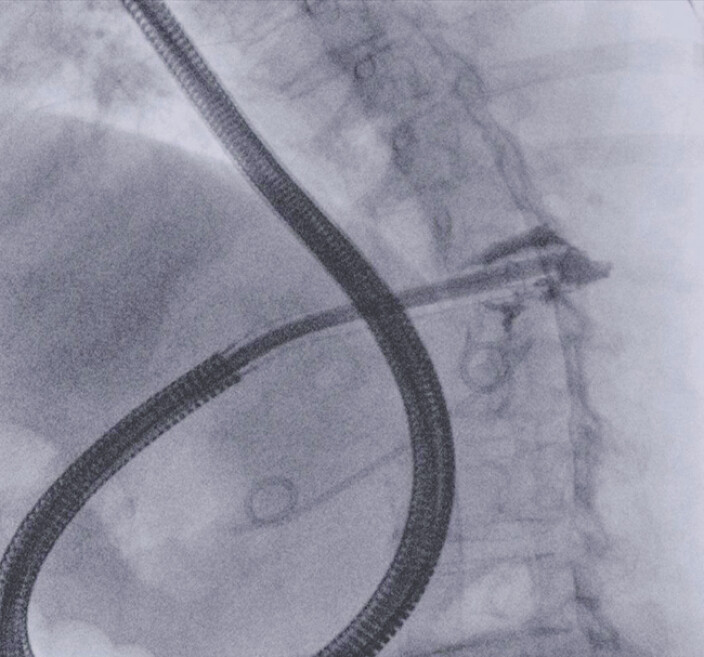
Scope suction during the procedure in order to facilitate necrosis removal.

**Fig. 3:**
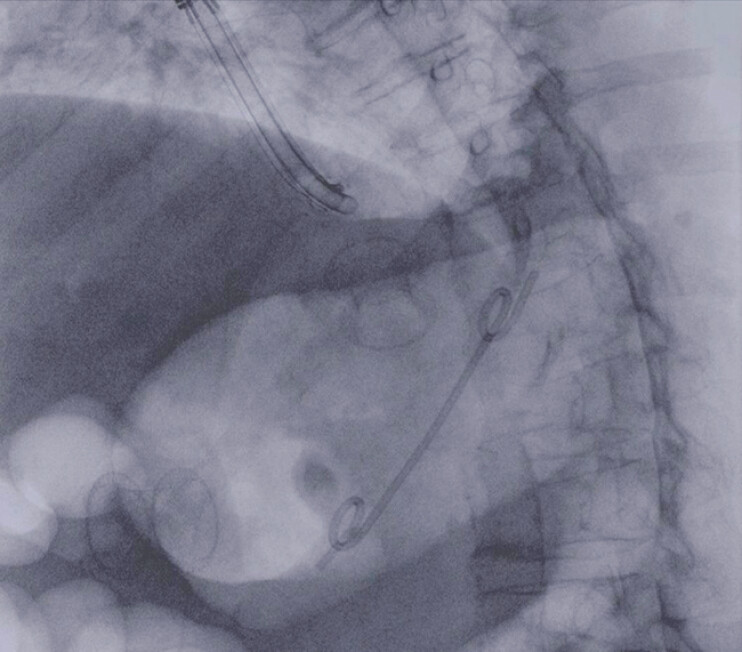
The large operative channel ensured efficient aspiration of substantial amounts of necrotic material.

Endoscopic ultrasound-guided drainage with placement of a lumen-apposing metal stent for a large infected walled-off necrosis. The sequence shows pneumatic dilation of the stent, endoscopic necrosectomy, and placement of two double-pigtail plastic stents with the stent-in-stent technique.Video 1

After 2 weeks, a computed tomography scan was performed, which confirmed the resolution of WON. The stent was then removed, and the patient was discharged without any complications.

Our case underlines the feasibility of performing endoscopic necrosectomy of an infected WON using a new single-use endoscope, theoretically avoiding the risk of scope infection 4 . Moreover, the large working channel (4.2 mm) may reduce procedure time and avoid multiple endoscopic sessions.

Endoscopy_UCTN_Code_TTT_1AS_2AJ

## Notes

Endoscopy E-Videos https://eref.thieme.de/e-videos
*E-Videos* is an open access online section of the journal *Endoscopy* , reporting on interesting cases and new techniques in gastroenterological endoscopy. All papers include a high-quality video and are published with a Creative Commons CC-BY license. Endoscopy E-Videos qualify for HINARI discounts and waivers and eligibility is automatically checked during the submission process. We grant 100% waivers to articles whose corresponding authors are based in Group A countries and 50% waivers to those who are based in Group B countries as classified by Research4Life (see: https://www.research4life.org/access/eligibility/ ). This section has its own submission website at https://mc.manuscriptcentral.com/e-videos .
